# Prognostic value of exercise echocardiography in patients with wild-type transthyretin amyloidosis

**DOI:** 10.48101/ujms.v127.8410

**Published:** 2022-03-28

**Authors:** Cristina Aguilera Agudo, Vanessa Moñivas Palomero, Esther González López, Susana Mingo Santos

**Affiliations:** ^a^Puerta de Hierro University Hospital of Majadahonda, Madrid, Spain; ^b^Heart Failure and Inherited Cardiac Diseases Unit, Department of Cardiology, Puerta de Hierro University Hospital of Majadahonda, CIBERCV, Madrid, Spain

**Keywords:** Wild-type transthyretin amyloidosis, contractile reserve, right ventricular free wall longitudinal strain

## Abstract

**Background:**

Wild-type transthyretin amyloidosis is a systemic disease with predominantly cardiac symptoms. The aim of this study was to assess the short-term prognosis of these patients through contractile reserve measured by stress echocardiography, given the usefulness that this parameter has demonstrated in other populations. We considered major events as death from any cause and hospitalization for heart failure.

**Material and methods:**

We conducted a study with a 1-year follow-up in 11 patients who were proposed to undergo a stress echocardiogram, with the follow-up as usual according to their doctor. We excluded pacemaker wearers, patients with permanent atrial fibrillation, those incapable of exertion at low loads, and those with poor acoustic windows.

**Results:**

We found that contractile reserve estimated by right ventricular free wall longitudinal strain is correlated with a lower rate of death (all of them cardiovascular deaths) and hospitalizations for heart failure.

**Conclusions:**

Contractile reserve assessed by right ventricular free wall longitudinal strain is a predictor of major events in patients with wild-type transthyretin cardiac amyloidosis.

## Introduction

Wild-type transthyretin amyloidosis with cardiac involvement (ATTRwt-CA) is an infiltrative and progressive disorder caused by the extracellular deposition of transthyretin amyloid fibrils in the heart in the absence of causal mutations. The prevalence of the disease increases with age, with nearly all affected patients over 60 years of age (1). Current studies suggest that ATTRwt-CA is an underdiagnosed cause of heart failure (HF), with a prevalence rate of 13% in a cohort of patients presenting with HF and preserved left ventricular ejection fraction (LVEF) (2). Non-invasive cardiac imaging techniques have boosted the recognition of this entity in recent years. Echocardiography is the cornerstone of the initial diagnosis of ATTRwt-CA (3), and speckle-tracking (ST) strain transthoracic echocardiography, especially left ventricular global longitudinal strain (LVGLS), has emerged as a sensitive and specific tool for its diagnosis (4). Nonetheless, the prognostic value of strain in ATTRwt-CA has not yet been well established, and data on the role of stress echo in cardiac amyloidosis are scarce.

## Materials and methods

We prospectively selected 11 ATTRwt-CA patients from our center database of 66 patients. Five of the 11 patients were diagnosed by endomyocardial biopsy and six of the 11 by suggestive clinical and echocardiographic data together with positive technetium-99m-3,3-diphosphono-1,2-propanodicarboxylic acid (99mTc-DPD) scintigraphy (Perugini uptake grade 3 or 4), negative serum-free light chains, negative serum, and urine immunofixation and genetic testing, demonstrating no mutations in transthyretin. We proposed to include them in our trial consisting of stress echocardiography if they met the inclusion criteria. There were no rejections to the inclusion in the trial. The study was approved by the ethics committee of our hospital. Patients were included prior to informed consent, if they had a diagnosis of ATTRwt-CA, excluding pacemaker wearers (15%), patients with permanent atrial fibrillation (AF) (44%), those incapable of exertion at low loads (6%), and those with poor acoustic windows or segmental alterations in the baseline echocardiogram (19%). All patients included had a mean New York Heart Association (NYHA) functional class of II-III.

Basal echocardiographic parameters were obtained according to the American Society of Echocardiography guidelines and standards (5). Stress echocardiography was performed using an upright bicycle ergometry protocol starting at a workload of 25 watts and increasing by 25-watt increments every 3 min. ST parameters, including right ventricular global longitudinal strain (RVGLS), free-wall right ventricular longitudinal strain (free-wall RVLS), and LVGLS, were analyzed using QLAB Philips 10.7 version (Philips Medical Systems, Best, the Netherlands). Contractile reserve (CR) was defined as a 2% increase over the basal LVGLS, RVGLS, and free-wall RVLS or an increase of 5% in LVEF (6, 7). The major cardiovascular (CV) events including death from any cause and HF hospitalizations during follow-up were captured for the analysis. No specific follow-up of the patients was performed because they were included in the study; however, it was established at the discretion of the treating doctor.

The distribution of all study variables was assessed using the Kolmogorov–Smirnov test, rejecting the assumption of normality when the significance reached in the test was <0.05. Categorical variables are presented as frequencies and percentages, and continuous variables are expressed as mean ± standard deviation (or median and interquartile range in those which do not fit the normal distribution). To calculate the differences between groups, the chi–square test was used to compare categorical variables and Student’s *t*-test for independent samples for continuous variables (or Mann–Whitney test if they do not fit a normal distribution). A value of *P* < 0.05 was considered statistically significant.

An event-free survival curve (Kaplan–Meier estimate) was performed by dividing the patients according to the presence or absence of CR in the stress echocardiogram, while the groups were compared using the log-rank statistical test.

## Results

Of the 11 patients included (mean age 77.6 ± 2.6 years; 91% male), only 2 (18%) achieved their predicted target heart rate during the stress echo. During a follow-up of 1.6 ± 0.1 years, six patients (54.5%, from now on: Group 1) presented major events: three deaths (27.3%) and six HF hospitalizations (54.5%). In patients with events (Group 1), the prevalence of CR estimated both by LVEF and especially by strain parameters related to the right ventricle (RV) was lower compared with Group 2 (patients without CV events) (Table 1 and 2).

**Table 1:** Echocardiographic parameters at rest and on exertion comparing ATTRwt patients with CV events during follow-up (Group 1) and those free of events (Group 2).

Variable	Group 1 *n* = 6	Group 2 *n* = 5	*P*
Septal thickness (mm)	18 ± 1.2	16.6 ± 0.7	0.17
Index LA volume (mL/m^2^)	52.6 ± 8.0	44.6 ± 6.0	0.23
Resting Lateral e´ wave (cm/s)	5.8 ± 0.7	9.9 ± 1.7	0.05
Resting Lateral E/e’ ratio	15.9 ± 1.8	9.0 ± 1.6	0.02
Resting LVEF (%)	54.8 ± 4.0	52.8 ± 2.5	0.58
LVEF on exertion (%)	45.3 ± 6.4	58.0 ± 2.8	0.14
CR by LVEF (%)	16.7	80	0.05
Resting LVGLS (%)	−13.0 ± 1.0	−16.1 ± 1.0	0.07
LVGLS on exertion (%)	−12.0 ± 1.8	−15.0 ± 1.6	0.07
CR by LVGLS (%)	16.7	40	0.41
Resting RVGLS (%)	−12.2 ± 1.1	−13.0 ± 1.3	0.71
RVGLS on exertion (%)	−9.5 ± 0.9	−17.3 ± 1.1	0.01
CR by RVGLS (%)	0	80	0.01
Resting free-wall RVLS (%)	−12.6 ± 1.2	−13.0 ± 1.4	0.65
Free-wall RVLS on exertion (%)	−8.8 ± 1.0	−18.3 ± 1.0	0.01
CR by free-wall RVLS (%)	0	100	0.002
Resting TAPSE (mm)	14 ± 2.0	17.8 ± 1.8	0.10
TAPSE on exertion (mm)	12.8 ± 1.6	19.2 ± 1.4	0.02
S´ velocity (cm/s)	8.4 ± 1.4	13.6 ± 0.6	0.05
Exercise time (s)	195 (167–360)	240 (217–785)	0.36

LA: left atrium; LVEF: left ventricular ejection fraction; CR: contractile reserve; LVGLS: left ventricular global longitudinal strain; RVGLS: right ventricular global longitudinal strain; Free-wall RVLS: free-wall right ventricular longitudinal strain; TAPSE: tricuspid annular plane systolic excursion.

**Table 2:** Overall survival according to CR by free-wall RVLS.

Patient	Event	Date of follow-up (days)
1	HF hospitalization and death	403
2	HF hospitalization and death	555
3	HF hospitalization	581
4	HF hospitalization	596
5	HF hospitalization	638
6	HF hospitalization and death	684

HF: heart failure.

**Figure 1:**
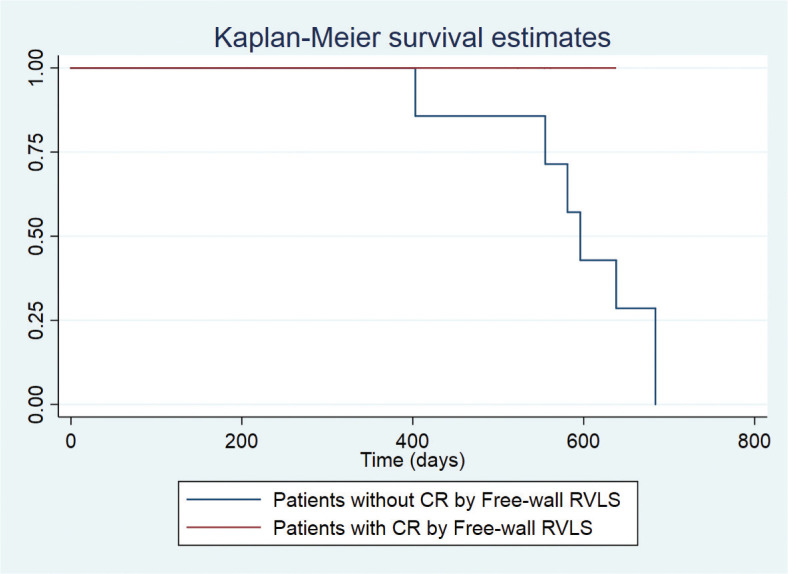
CR: contractile reserve, Free-wall RVLS: free-wall right ventricular longitudinal strain.

Group 1 was significantly older and had significantly higher left ventricle (LV) filling pressures at rest, assessed by mitral E/e’ ratio at the lateral annulus. The presence of CR estimated by LVEF was lower in Group 1. Both RVGLS during exercise and CR assessed by this parameter were significantly higher in Group 2. Similarly, free-wall RVLS during exercise and CR estimated by this parameter were higher in Group 2 (−8.8 ± 1.0 vs. −18.3 ± 1.0, *P* = 0.01; 0% vs. 100%, *P* = 0.002). CR assessed by free-wall RVLS emerged as a predictor of short-term survival free of death and HF hospitalizations with a log-rank p-value by Mantel-Cox of 0.034 (Figure 1). In addition, Group 2 was found to have a better RV function measured by the tricuspid annular plane systolic excursion (TAPSE) and annular systolic tissue Doppler imaging velocity on exertion compared with Group 1.

## Discussion

The echocardiogram, especially the estimation of LVEF, is the main prognostic factor for patients with HF, indicating the initiation of pharmacological treatment or even implantation of cardioverter-defibrillator implantation (8). ST was used to go a step further and try to predict less evident or advanced dysfunctions. This may be a way to avoid further deterioration if we treat patients earlier. The CR is proved especially useful in patients with valvular heart disease (mainly aortic stenosis), and it is observed as a good prognostic factor, indicating surgery in patients with reduced LVEF, low flow, and low-gradient symptomatic aortic stenosis (9). It was also used for diagnosis in patients with risk of CV toxicity due to chemotherapy and for initiation of preventive therapy against this toxicity (10). Taking into account the use of ST as an early marker of LV dysfunction, as well as its prognostic value, we propose to apply it to patients with ATTRwt-CA, as estimating it through a stress test involves a low risk for the patient.

To our knowledge, this is the first prospective cohort of patients with ATTRwt-CA evaluating the prognostic value of an exercise test in this population. According to the study results, stress echocardiography with strain analysis has an additional value in the identification of patients at risk of CV complications, with the presence of CR assessed by free-wall RVLS, resulting in a predictor of short-term survival events. Moreover, patients without events showed better values of RV echocardiographic parameters, especially with exercise. The measurement of RV function could be of great value in the prognostic evaluation of ATTRwt-CA patients, guiding and helping decide on the initiation of specific treatment for this disease. Further prospective and larger studies to confirm these results are needed.

Although this is a study with a small number of patients, which limits its application in clinical practice, and only a univariate analysis was performed, we believe that it can serve as a reference for the study of the value of this parameter in new series. We recognize as main limitations, besides the low sample size, the fact that even within our target population, many patients (83%) were excluded mainly due to AF, which is an important consideration for its daily clinical application. Moreover, there is no defined limit for the definition of CR, so it is variable in the literature. It is also noteworthy that the decrease in both LVEF and strain with exercise in these patients is contrary to normal physiological findings. This suggests, without being able to exclude errors in the measurement of these parameters as they were performed by experts in stress echocardiography, that there is a basal myocardial involvement even with normal LVEF that could justify the poor tolerance to exercise in this population. However, given that establishing the prognosis of these patients can be difficult in some cases, we consider this test to be objective data to establish the short-term prognosis in this disease or even justify the initiation of treatment specifically directed against ATTRwt-CA. We highlight the fact that despite the age of these patients, all the deaths recorded were of CV causes, mainly due to HF.

To conclude, stress echocardiography parameters could add a prognostic value in ATTRwt-CA, and free-wall RVLS CR appears to be a good predictor of event-free survival in this group of patients.
